# Hydrogels for Osteochondral Interface Regeneration: Biomaterial Types, Processes, and Animal Models

**DOI:** 10.3390/gels12010024

**Published:** 2025-12-27

**Authors:** Sanazar Kadyr, Bakhytbol Khumyrzakh, Swera Naz, Albina Abdossova, Bota Askarbek, Dilhan M. Kalyon, Zhe Liu, Cevat Erisken

**Affiliations:** 1 Department of Chemical and Materials Engineering, School of Engineering and Digital Sciences, Nazarbayev University, Astana 010000, Kazakhstan; sanazar.kadyr@nu.edu.kz (S.K.); bakhytbol.khumyrzakh@nu.edu.kz (B.K.); swera.naz@nu.edu.kz (S.N.); albina.abdossova@nu.edu.kz (A.A.); 2 Department of Biology, School of Sciences and Humanities, Nazarbayev University, Astana 010000, Kazakhstan; bota.askarbek@nu.edu.kz; 3 Chemical Engineering and Materials Science, Stevens Institute of Technology, Hoboken, NJ 07030, USA; 4 Academy of Medical Engineering and Translational Medicine, Tianjin University, Tianjin 300072, China; zheliu@tju.edu.cn; 5 Biomedical Engineering Program, School of Engineering and Digital Sciences, Nazarbayev University, Astana 010000, Kazakhstan

**Keywords:** osteochondral, hydrogel, biomaterial processing, 3D printing, extrusion, animal models, regeneration, regenerative medicine, tissue engineering

## Abstract

The osteochondral interface (OCI) is a structurally and functionally complex tissue whose degeneration or injury often results in poor healing and joint dysfunction due to its avascular and hypocellular nature. Conventional surgical treatments remain suboptimal, prompting growing interest in regenerative approaches, particularly with the utilization of hydrogel-based biomaterials that can mimic the extracellular matrix and support osteochondral regeneration. This study reviewed types of hydrogels, scaffold processing techniques, and animal models for OCI regeneration. Our search demonstrated that gelatin, alginate, chitosan, and hyaluronic acid were the most frequently investigated hydrogels. Layered constructs dominated current scaffold designs, while advanced methods such as 3D printing and extrusion demonstrated unique potential to create graded architectures resembling the native OCI. Rabbits were the most widely used in vivo models, though translation will require larger animal studies with clinically relevant defect sizes. Future efforts should focus on developing mechanically reinforced, biologically active, and continuously graded hydrogels, supported by standardized preclinical validation in large-animal models, to accelerate translation toward clinical solutions for osteochondral regeneration.

## 1. Introduction

The osteochondral interface (OCI) refers to the graded region between the cartilage layer and the underlying bone tissue in articulating joints [1]. The OCI plays an important role in providing mechanical stability to the joint and preventing the vascularization and mineralization of articular cartilage [2,3]. OCI lesions generally occur due to age and trauma, and exhibit a high prevalence of high-impact sports, including football, basketball, and soccer [4]. Additionally, if not treated, progression of cartilage degeneration in the joints can lead to OCI degeneration, which may result in complete dysfunction of the joint. Due to its avascular and hypocellular nature, injuries related to articular cartilage do not heal themselves and require surgical treatments, including but not limited to microfracture, mosaicplasty, and subchondral drilling [2]. Despite reported encouraging results, currently available treatment options seem to have far from optimal outcomes, and therefore, alternative strategies are being investigated [5]. Despite growing research on the OCI, its pathogenesis and natural history are not fully understood. Engineering the OCI using regenerative approaches has the potential to offer a solution; however, our recent review on the topic [2] demonstrated a need for a more detailed search on the use of hydrogel-based biomaterials, processing techniques, and animal models. This is essential because a systematic approach to OCI regeneration requires utilization of a suitable biomaterial, an advanced technology to process the biomaterials to mimic the target structure, and a relevant in vivo model as a minimum.

Hydrogel scaffolds are essential in tissue engineering because they provide a 3D framework for cell adhesion, growth, and nutrient exchange while also mimicking the mechanical properties of native tissue, ensuring flexibility and strength under joint stresses [6]. Due to their biocompatibility and biodegradability, they degrade into non-toxic products in a harmonized manner as the tissue regenerates. Both synthetic and natural biodegradable polymers have been widely explored, with natural materials such as alginate, gelatin, chitosan, hyaluronic acid, and collagen attracting special attention for their ability to replicate the extracellular matrix [7]. Alginate, a hydrophilic polysaccharide composed of mannuronic and guluronic acids, is valued for its low cost, availability, and scaffold-forming ability [7]. Gelatin is a biodegradable denatured collagen, naturally bioactive, and stable at body temperature once chemically crosslinked. Chitosan offers extracellular-matrix-like structure, biocompatibility, and antibacterial properties [8], while hyaluronic acid, a major component of synovial fluid, is used for its swelling capacity and biocompatibility, often modified for crosslinking in composite scaffolds [9]. Collagen-based hydrogels reinforced with nanomaterials enhance strength and can incorporate drugs such as ibuprofen, improving osteochondral repair strategies [10].

OCI tissue exhibits a graded design in its structure, composition, and function at the bone-cartilage transition [2,11]. Through the integration of diverse fabrication technologies, research teams have generated a robust portfolio of functionally graded materials for complex tissue interface regeneration including OCI tissue. One of the earliest foundational contributions was the development of hybrid twin-screw extrusion and electrospinning methodologies [11,12,13,14,15]. The twin-screw extrusion process facilitated effective mixing and melt processing, while the downstream electrospinning step allowed for the formation of nanofibrous meshes with tunable composition and structure. Additional efforts focused on the rheological tuning of processing conditions to optimize scaffold morphology and composition [16,17]. More recently, melt electrowriting was implemented to fabricate precisely architected and functionally graded substrates with micrometer-scale fidelity [18]. However, the general practice in fabricating tissue engineering scaffolds mostly relies on a layered approach, undermining the gradual change in its characteristics. Therefore, revealing the trend in the use of biomaterials and scaffolds will shed a light on the approaches used for biomaterial selection and processing, which can open new avenues for the treatment of OC-related defects.

Animal models play a critical role in tissue engineering research by providing a physiological environment similar to the intended use in which the safety, functionality, and regenerative potential of biomaterials and engineered constructs can be evaluated before clinical translation. Unlike in vitro systems, which are limited in their ability to replicate the complexity of living tissues, animal models allow researchers to study host–biomaterial interactions under dynamic physiological conditions, including immune responses, vascularization, mechanical loading, and long-term integration. Small animals such as mice [19] and rats [20] are widely used for early-stage testing due to their availability, low cost, and suitability for mechanistic studies, although their joint size and biomechanical properties differ substantially from humans. Larger animals, including rabbits [21,22], sheep [23], pigs [24,25], and horses [26,27], provide more clinically relevant models by offering defect sizes, cartilage thickness, and load-bearing environments closer to those of human joints [28]. Despite ethical considerations, intentions to phase out the use of animals in research, and cost limitations, animal models still remain essential in bridging the gap between bench-scale innovations and human application, ensuring that novel tissue engineering strategies are both effective and safe for eventual clinical use.

The OC-related research needs to be systematically identified to clearly see the current status quo to make plausible recommendations for better regenerative outcomes. Additionally, given the diversity of hydrogel chemistries and fabrication methods, this analysis helps identify underexplored combinations and methodological gaps critical for translational success. Therefore, this study aims to perform a literature review on OCI regeneration in order to determine the most widely employed hydrogels, processing techniques utilized for shaping these biomaterials, and their use in animal models.

## 2. Hydrogel Biomaterials for OCI Regeneration

Characterization of the biomechanical and rheological properties of the cartilage and the underlying subchondral bone demonstrated the viscoelastic nature of the interface exhibiting a gel behavior [29]. The gel behavior of the osteochondral tissue, apparently, motivated the researchers to use hydrogels as a biomaterial for its regeneration. Hydrogels have emerged as promising candidates for mimicking the OCI due to their high water content, structural similarity to native extracellular matrix (ECM), and capacity for biochemical and mechanical tunability. Their viscoelastic nature allows for adjustment of stiffness and degradation kinetics to approximate the gradient properties across the cartilage-to-bone transition. Additionally, their porous network facilitates nutrient diffusion and can serve as a reservoir for bioactive molecules, growth factors, and cells to promote region-specific tissue regeneration. Despite these advantages, several challenges remain that limit their translational potential. Poor interfacial integration with native subchondral bone and cartilage often results from insufficient mechanical anchorage or mismatched degradation rates, leading to delamination or incomplete tissue bonding [30]. Additionally, uncontrolled swelling and degradation can compromise structural stability, particularly under physiological loading conditions. Moreover, achieving secure fixation during implantation remains difficult due to the compliant and hydrated nature of the material. To overcome these limitations, recent strategies such as incorporating reinforcing nanofillers, developing gradient or composite hydrogel systems, and employing biofunctionalization approaches to enhance cell adhesion and matrix deposition are being actively investigated [31,32] to improve both mechanical integrity and biological integration at the OCI.

To elaborate the use of hydrogel biomaterials in OCI repair and regeneration, we searched the literature from the last 25 years and organized the findings in Table S1. Notably, gelatin-based hydrogels were the most extensively used biomaterial [21,32,33,34,35,36,37,38,39,40,41,42,43,44,45,46,47,48,49,50,51,52,53,54,55,56,57,58,59,60,61,62,63,64,65], followed by alginate-based hydrogels [11,20,22,23,24,65,66,67,68,69,70,71,72,73,74,75,76,77,78] and chitosan [27,79,80,81,82,83,84,85,86,87,88,89,90,91]. Hyaluronic acid [92,93,94,95,96,97,98,99,100], polyvinyl alcohol [101,102,103,104,105,106,107,108,109,110], oligo(poly(ethylene glycol) fumarate) [25,111,112,113,114,115,116,117,118], and poly(ethylene glycol) [119,120,121,122,123,124,125,126] were also notable, particularly for their versatility in forming hybrid scaffolds. Gellan gum [127,128,129,130,131], silk fibroin [31,132,133,134,135,136], agarose [137,138,139], poly-(2-Acrylamido-2-methylpropanesulfonic acid)/poly-(N,N′-dimethyl acrylamide) (PAMPS/PDMA) [140,141,142], collagen [143,144], chondroitin sulfate [145,146], polyacrylamide (PAAm) [147,148], polyaminoacid (PAA) [149], poly(N-acryloyl glycinamide)/[tris(hydroxymethyl)methyl]acrylamide (PNAGA/THMMA) [150], poly(L-glutomic) acid-phenylboronate ester (PLGA-PBE) [151], polyglucosamine (PG) [152], elastin-like recombinamer (ELR)-based [153], and heparin-conjugated fibrin (HCF) [154] were also used.

Gelatin-based systems were the most frequent, reflecting a preference for natural polymers that provide intrinsic bioactivity and are already cleared for biomedical use [155]. Gelatin, a denatured derivative of collagen, provides inherent cell-adhesion motifs that facilitate chondrocyte and osteoblast attachment and proliferation [43]. Its natural biodegradability and tunable crosslinking behavior make it an ideal base material for osteochondral applications; however, its relatively weak mechanical strength and rapid degradation often require reinforcement with synthetic polymers or nanoparticles [38].

Alginate, a polysaccharide derived from brown algae, is widely used because of its mild gelation through ionic crosslinking with divalent cations, cytocompatibility, and ease of forming 3D scaffolds. Despite its excellent biocompatibility, alginate lacks intrinsic cell-adhesive domains, which can limit tissue integration unless modified with peptides or blended with proteins such as gelatin [37].

The chitosan-based hemostatic dressings are on the market, while the injectable scaffolds and drug delivery systems are still under investigation [156]. Chitosan, obtained from chitin deacetylation, offers antibacterial activity, hemostatic properties, and a structural similarity to glycosaminoglycans in cartilage ECM [81], making it suitable for osteochondral defect repair. Nonetheless, its poor solubility and pH sensitivity can affect reproducibility and stability [157].

Hyaluronic acid (HA) plays a critical role in modulating cell signaling, promoting chondrogenesis, and enhancing viscoelastic properties of the constructs. It also contributes to the recruitment and differentiation of mesenchymal stem cells toward the chondrogenic lineage [92]. The main limitation of HA-based hydrogels lies in their mechanical weakness and susceptibility to enzymatic degradation, which necessitates crosslinking or combination with more robust polymers or fillers [95,96].

Synthetic systems such as PEG [122], OPF [118], and PVA [109] also gained interest, often as hybrid components to provide tunable mechanical properties and degradation kinetics. Silk fibroin and collagen, despite being natural biomaterials, have not been frequently used alone due to their incompatibility in terms of mechanical properties.

It should be noted that publication frequency does not necessarily reflect superior biological or mechanical performance of hydrogels and that functional effectiveness should be taken as the key indicator of success. In this context, the studies that appeared in this search demonstrate expression of relevant markers and relevant ECM production in respective zones, defect filling, and scaffold–tissue integration. However, a previous literature review [2] revealed that tidemark, an important component of the OC tissue, is not formed. Realizing this gap, recent studies focused on the design and fabrication of OC scaffolds with a tidemark component [11]. If tidemark is not formed, the blood vessels from the subchondral bone can penetrate the articular cartilage region to eventually form fibrocartilage tissue, which is inferior to hyaline cartilage. Evaluation of the in vivo outcomes of the list of publications searched here in terms of quality of regeneration was not performed and it should be considered as a limitation of this study.

Overall, these natural polymers dominate osteochondral regeneration research because they are inherently biocompatible, support cell–matrix interactions, and can be modified for desired mechanical and biological performance. Particularly, the use of these hydrogel biomaterials to form layered structures that mimic the structure of the OCI should be noted. In this regard, our findings demonstrate a clear trend from simple, layered hydrogels toward multifunctional and structurally graded constructs that incorporate characteristics of the native tissue to actively direct osteochondral regeneration. Despite these advances, challenges remain in achieving sufficient load-bearing strength, an inherent property of the hydrogels, and the required long-term integration. Future perspectives emphasize the development of mechanically reinforced and continuously graded hydrogels, integration of immunomodulatory and spatiotemporal bioactive signaling, and adoption of standardized large-animal preclinical models to bridge the gap toward clinical application.

## 3. Techniques Used to Process Hydrogels

The techniques employed for the processing of each biomaterial type to generate homogeneous (H), layered (L), and graded (G) structures are organized in Table 1.

Processing techniques are employed to shape and structure biomaterials for their ultimate use as scaffolding materials and are critical for creating functional materials for osteochondral interface regeneration. Clearly, 3D printing appears as the most frequently used technique to process biomaterials for OC regeneration over the years. This was followed by casting, freeze-drying, molding, and injection. Electrospinning, salt leaching, implantation, and extrusion processes contributed to a lesser extent. These techniques were used to process biomaterial to create uniform, layered, and gradient structures. Among these, layered structures appear as the most frequently used structure for OCI regeneration, followed by homogeneous and graded.

3D printing enables precise spatial control and multi-material deposition (depending on the number of nozzles that could be installed on the printer), which makes it especially suitable for layered [56] and graded [78] designs. However, 3D printers lack the mixing capability for the ingredients during processing, particularly important when solids or biomolecules are incorporated into the mixture to create heterogeneous structures seen in OC interface [32,43,45,51]. Therefore, despite their significant advantage in creating precise shapes, 3D printers still require improvement in terms of dispersion and distribution of the ingredients while processing the biomaterials continuously [158].

Casting and molding remain common due to their simplicity and cost-effectiveness, but they mainly produce homogeneous or bi-layered constructs with limited ability to replicate the gradient seen in the native tissue [54,90]. There have been some attempts to create graded structures using casting [102,129] and molding [23,34,69,75]; however, these likely lack some of the important features of continuous processes including continuous mixing of the ingredients.

Freeze-drying yields highly porous scaffolds that support nutrient transport and cell ingrowth, although the process typically generates random, mechanically weak architectures rather than controlled gradients. Despite these, this process has been used to create graded structures using alginate [76], HA [100], OPF [118], and PVA [107].

Injection is particularly useful for injectable hydrogels, offering defect-fitting scaffolds for minimally invasive delivery [58], though these constructs are generally homogeneous unless specifically designed for multiphase systems.

Examples to create graded scaffolds include gelatin [48] and hyaluronic acid (HA) [92]. Less frequently employed approaches also contribute important capabilities. For example, electrospinning generates nanofibrous scaffolds resembling collagen fibrils [157,159] but is limited in the thickness of the scaffold [14,160]. It enables creating gradient if parameters are selected properly [14]. Salt leaching provides controlled pore diameter and porosity determined by the size distribution of the porogen, and can generate graded structures when combined with other techniques such as extrusion [17]. Notably, extrusion, though used rarely, holds great potential because it allows continuous co-extrusion of multiple materials and the creation of compositional or structural gradients that closely resemble the native OCI [12,14]. By precisely controlling the respective feed rates of the ingredients and the mixing parameters during deposition, extrusion can generate seamless transitions in mineral content, stiffness, or bioactive cue distribution, which layered approaches fail to achieve. Despite its underutilization in the studies evaluated here, extrusion represents a promising direction for advancing scaffold design toward more biomimetic and mechanically competent constructs.

Overall, while 3D printing dominates due to architectural control, extrusion uniquely enables compositional gradients. Future hybrid systems combining both could resolve current limitations. Regarding the structure of the scaffold, while layered scaffolds currently dominate, followed by homogeneous and graded, the strategic integration of extrusion with other fabrication methods could accelerate the development of functionally graded scaffolds that better replicate the native osteochondral interface and improve translational outcomes.

The fabrication methods that appeared in published studies have both strengths and limitations for the scaffold’s biological, mechanical, and translational suitability. In this regard, different fabrication techniques impart distinct structural, mechanical, and biological characteristics to hydrogel-based scaffolds, which ultimately influence their translational suitability. 3D printing (additive manufacturing) offers precise spatial control over geometry and composition, enabling gradient architectures that mimic the osteochondral transition. It allows integration of multiple materials and bioactive cues, leading to improved biological specificity and reproducibility. However, printing resolution and material viscosity constraints can limit mechanical robustness and scalability. 3D printing enables the investigators to combine multiple materials, including biologicals, to create biological structures similar to the native OC interface and tune mechanical properties as needed. These would allow for the formation of clinically relevant scaffolds. Casting is a simple, cost-effective approach suitable for forming bulk hydrogels with uniform properties but it lacks control over microarchitecture and gradient formation, limiting its capacity to reproduce native tissue heterogeneity. Therefore, this technique lacks the capacity to create biological structures like native OC interface and formation of clinically relevant scaffolds. Freeze-drying produces highly porous scaffolds with interconnected networks conducive to cell infiltration and nutrient diffusion; however, the resulting structures often exhibit poor mechanical strength and require post-processing for load-bearing applications. Molding enables reproducible shaping and is compatible with various hydrogel systems, yet it provides minimal control over internal architecture and mechanical gradients, thus limiting the creation of native-like structures. Injection molding facilitates minimally invasive delivery and conformal defect filling in vivo, enhancing translational relevance, though it is restricted to shear-thinning or in situ crosslinkable hydrogels and may result in limited interfacial integrity. Electrospinning allows the creation of fibrous architectures that mimic ECM morphology, enhancing cell attachment and anisotropic mechanical properties, but the dense fiber packing can restrict cell infiltration and nutrient exchange. Additionally, due to the formation of highly porous meshes, the mechanical properties are usually inferior to those of the native OC tissue. Salt leaching produces macroporous structures with tunable pore size; however, residual porogens and limited reproducibility can compromise biocompatibility and mechanical uniformity. Lastly, extrusion provides scalability and moderate control over structure while maintaining cell viability during processing, but it may induce shear stress on encapsulated cells and yield constructs with relatively low resolution compared to 3D printing. Therefore, extrusion enables the investigators to combine multiple materials, including biological, to create biological structures similar to the native OC interface and tune mechanical properties as needed, thus allowing for the formation of clinically relevant scaffolds. Overall, combining complementary fabrication methods (e.g., 3D printing or extrusion with electrospinning or freeze-drying) can help balance biological performance, mechanical functionality, and translational feasibility, advancing the development of clinically relevant osteochondral hydrogel systems. The authors previously combined the process of electrospinning with extrusion to increase the degree of freedom to create gradients in the scaffold to mimic the native OC interface [12,14,15]. Similarly, the extrusion method was combined with salt leaching to create gradients of porosity to mimic the structure of the bone [17]. The findings clearly demonstrated that combining multiple processes was very useful to generate scaffolds structurally and functionally similar to native tissues.

## 4. Animal Models for OCI Interface Regeneration

Animal models serve as a relevant environment for trying the biomaterials in the context of translational research. However, it is generally challenging to find the right animal model for the specific research direction considering limitations associated with ethics, physiological similarity, cost, and the availability of suitable breeds. Small animals are usually less costly yet lack physiological similarity, while large-animal models are physiologically more relevant yet costly and not easily available as laboratory animals.

A summary of the search is given in Table 2 in terms of defect anatomical location, defect size, duration of in vivo study, and the methods used to test the quality of the outcome for each animal model used.

The compiled data across multiple animal models highlights the diversity of preclinical osteochondral defect studies in terms of defect size, location, in vivo duration, and outcome assessment methods. Small-animal models are typically used for early-stage investigations due to their small defect sizes 2-3 mm) and short in vivo durations 6-24 weeks), allowing rapid evaluation of cellular or biomaterial-based interventions with histology, immunohistochemistry (IHC), and micro-CT analysis [5,7,12,14,18,22,27]. Rabbits serve as an intermediate model with moderate defect sizes (2–6 mm) and similar follow-up periods (4–24 weeks), providing a balance between practicality and translational relevance, frequently assessed via histology, IHC, macroscopic scoring, and imaging [1,3,6,9,13,15,19,21,24,28]. Larger animal models, including pigs, dogs, sheep, and horses, offer defects that more closely mimic human joint dimensions (4–10 mm) and extended in vivo durations (12–48 weeks), enabling comprehensive biomechanical testing, advanced imaging, and tissue integration analyses [2,4,8,10,11,16,17,20,23,25,26]. These models are particularly valuable for translational studies, although they require greater resources, longer study times, and complex ethical considerations. Overall, this compilation underscores the importance of selecting an animal model that balances experimental feasibility with translational relevance, while also providing a reference framework for interpreting defect size, healing duration, and methodological approaches across species.

**Figure 1:**
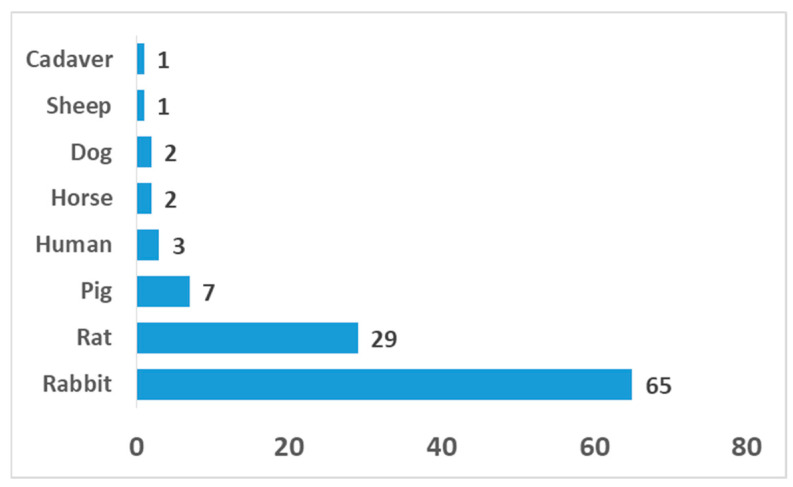
Frequency of animal models utilized for in vivo evaluation of hydrogel-based osteochondral regeneration strategies.

The distribution of animal models is shown in Figure 1. As expected, rabbits were the most frequently used species in OCI-related research mainly because of their availability, cost-effectiveness, and physiological relevance, i.e., the joint sizes suitable for creating osteochondral defects [79,93].

Rats, the second most common choice, are less costly but lack physiological similarity in terms of defect size [95]. Large animals such as pigs [24,25], sheep [23], dogs [81], and horses [26,27] were rarely used, mainly in translational studies where load-bearing conditions and defect sizes are expected to match the human joint [28]. The distribution observed reflects a preference for small-animal models in early-stage evaluation due to clinical, practical, and ethical considerations, while large animals are reserved for preclinical research requiring biomechanical and clinical relevance. However, ethical and translational barriers related to large animals are worth noting here. The primary ethical barrier revolves around the widely accepted understanding that large animals are sentient beings capable of experiencing pain, distress, and suffering. Research protocols must rigorously adhere to principles designed to minimize harm, which is a significant moral burden on researchers and institutions. Additionally, the use of large animals, especially non-human primates, can evoke strong public opposition and mistrust. This social scrutiny influences funding decisions, regulatory policies, and the overall sustainability of research involving these species. Regarding the translational barriers, despite the anatomical and physiological similarities to humans that make large animals attractive as models, significant species differences exist in genetics, immune responses, drug metabolism, and disease pathology. This fundamental discordance often leads to promising results in animals that fail to translate to human clinical benefits as a result of high failure rates in clinical trials. Overall, small-animal models are widely used to demonstrate biological feasibility and early regenerative responses in osteochondral repair. However, their higher intrinsic healing capacity, reduced joint loading, and thinner cartilage structure limit direct translation to large-animal models and humans. As a result, outcomes observed in small animals may overestimate regenerative efficacy, underscoring the need for validation in large-animal models prior to clinical translation.

## 5. Authors’ Views: Emerging Trends, Technological Gaps, and Requirements for Translational or GMP Readiness

The field of osteochondral regeneration has undergone rapid development over the past two decades, with hydrogel-based biomaterials emerging as leading candidates for mimicking the unique structural and biochemical features of the OCI. This finding underscores not only the scientific maturity of the field but also an increasing recognition of the need to bridge laboratory research with regulatory and manufacturing frameworks suitable for clinical translation.

A notable direction in recent work is the design of multifunctional hydrogels that balance mechanical reinforcement, biochemical functionality, and structural fidelity. Traditional hydrogels such as gelatin, alginate, chitosan, and hyaluronic acid remain dominant; however, there is a clear shift toward hybrid and composite systems that incorporate nanoparticles, ceramics, or synthetic polymers to enhance mechanical integrity and durability. These modifications aim to overcome one of the long-standing barriers to clinical use, i.e., the inherently low load-bearing capacity of hydrogels, while maintaining their biocompatibility and tunable degradation profiles. Simultaneously, bioactive functionalization through the inclusion of growth factors or peptide motifs is gaining momentum, improving cell recruitment and guiding osteochondral differentiation.

Advanced fabrication strategies, particularly 3D printing, extrusion-based bioprinting, and electrospinning, have also emerged as transformative tools for creating graded or layered architectures that more closely replicate the native OCI. These technologies allow for spatial control of composition and stiffness, enabling more faithful modeling of the osteochondral gradient from cartilage to subchondral bone. However, ensuring batch-to-batch reproducibility, sterility, and mechanical consistency during manufacturing remains a major challenge, particularly as production scales up for translational and clinical applications.

Despite these advances, the translational pathway for hydrogel-based constructs remains hindered by the lack of standardized preclinical validation protocols and clear regulatory alignment with Good Manufacturing Practice (GMP) requirements. Validation protocols for osteochondral biomaterials should include harmonized mechanical testing (e.g., compressive modulus, fatigue resistance), degradation kinetics, and cell–material interaction studies under physiologically relevant conditions. Moreover, in vivo validation must evolve beyond small-animal models. While rabbits dominate current studies due to cost and accessibility, these models often fail to replicate the biomechanical environment of the human joint. Larger animal models—such as sheep, pigs, or dogs—are increasingly recognized as essential for assessing integration, mechanical durability, and long-term remodeling within clinically sized defects. The use of standardized defect geometries, consistent outcome measures, and long-term follow-up will be critical to ensuring cross-study comparability and regulatory acceptance.

From a regulatory standpoint, GMP readiness requires that hydrogel fabrication, crosslinking, sterilization, and packaging processes are well-controlled, documented, and reproducible. Materials must comply with ISO 10993 biocompatibility standards [161], and each step of production should adhere to GMP-compliant procedures, including cleanroom fabrication, validated sterilization, and rigorous endotoxin testing. The development of hydrogels using reagents and methods amenable to GMP production—such as enzyme-mediated crosslinking or photo-crosslinking with approved initiators—will greatly facilitate clinical transition. Furthermore, early dialogue with regulatory bodies can help align preclinical testing strategies with expectations for Investigational New Drug (IND) or Investigational Device Exemption (IDE) submissions.

Additionally, it is also worth providing structural properties of some of the products that moved to the translational phase. The CartRevive™ scaffold is a relatively new monophasic scaffold. It is a blend of dextran and hyaluronic acid conjugates. The CartRevive™ implant is undergoing human clinical trials to treat mild cartilage defects but not full-thickness OC defects. ChonDux™ is an adhesive hydrogel composed of photocrosslinkable polyethylene glycol with functionalized chondroitin sulfate. During clinical trials, the application of ChonDux™ was paired with debridement and microfracture, which breaks up the tidemark. MaioRegenTM Prime is a triphasic scaffold that aims to treat lesions in patients with Grade 4 osteoarthritis. A clinical study was conducted in 2017; however, patients had hypertrophic cartilage, cysts, edemas, sclerosis of the subchondral bone, and cleft formation in the repaired tissue. The triphasic scaffold was unable to regenerate the interface due, possibly, to the absence of a tidemark membrane. TruFit^®^ is a biphasic bioresorbable plug that targets full-scale osteochondral defects. A clinical study in which TruFit^®^ plugs were implanted in 10 patients reported that full integration was observed in only 3 patients at the 2-year follow-up. Other clinical studies have reported the formation of fibrous scar tissue, probably due to employing arthroplasty or mosaicplasty after implantation of the TruFit^®^ plug. ChondroMimetic^®^ is a biphasic scaffold. In a clinical study, the scaffolds were implanted at mosaicplasty donor sites, which allowed for vascular invasion. Hyalograft^®^ C is a modified hyaluronic acid membrane seeded with autologous chondrocytes and was introduced by Fidia Advanced Biopolymers in 1999. BST-CarGel^®^ is composed of chitosan and forms a hydrogel under physiological conditions. It is used together with microfracture. CARTISTEM^®^ is a hydrogel composed of MSCs and hyaluronic acid for patients with severe knee osteoarthritis. CaReS^®^ is a collagen type 1 implant with autologous chondrocytes. NeoCart^®^ is a collagen type 1 implant for treating chondral defects in patients with ICRS grade 3 osteoarthritis. Cartipatch^®^ is a hydrogel plug for osteochondral defects comprising autologous chondrocytes suspended in an alginate–agarose mixture.

## 6. Conclusions

This review highlights the growing role of hydrogel-based biomaterials and scaffold processing strategies in osteochondral interface regeneration. First, hydrogels are good candidates for osteochondral regeneration and should be continued. Gelatin, alginate, chitosan, and hyaluronic acid emerged as the most widely investigated hydrogels. These could be complemented by the addition of biologically active molecules and the design and fabrication of functionally graded scaffolds to better mimic the structure/function of the native osteochondral interface. Second, processing techniques should be optimized to create biomimetic structures according to the needs of the orthopedic research community. In this regard, extrusion and 3D printing should be combined for better outcomes. Third, rabbits continue to serve as the most common animal model, though large-animal studies are essential to bridge the translational gap. Large animals are physiologically more relevant and should be made available for research at more affordable costs. Furthermore, research funding sources should initiate more calls related to the design and fabrication of biomimetic scaffolds to accelerate osteochondral-related research outputs to address the needs of patients.

## Figures and Tables

**Table 1:** Processing techniques employed for each biomaterial type.

	3D Printing			Casting			Freeze-Drying			Molding			Injection			Electrospinning			Salt Leaching			Implant			Extrusion		
Hydrogel	H	L	G	H	L	G	H	L	G	H	L	G	H	L	G	H	L	G	H	L	G	H	L	G	H	L	G
Gelatin	2 [21,60]	9 [35,36,42,44,47,50,53,56,59]	6 [32,43,45,51,62,78]		8 [37,38,39,41,46,49,54,57]			1 [40]		1 [61]	2 [63,64]	1 [34]	4 [33,55,58,65]		1 [48]												
Alginate	1 [72]	1 [68]	5 [11,20,70,72,77]	3 [22,66,67]					1 [76]		2 [73,74]	3 [23,69,75]		1 [24]							1 [71]						
Chitosan	2 [83,85]						3 [81,88,89]	4 [40,84,87,91]		1 [90]	2 [80,86]					3 [27,79,82]											
HA		2 [96,97]			3 [95,98,99]				1 [100]		1 [94]		2 [93]		1 [92]												
OPF				1 [25]	2 [113,117]		1 [116]		1 [118]	2 [112,114]	2 [111,115]																
PEG	1 [121]	2 [125,126]		1 [122]	1 [120]						1 [119]	1 [123]		1 [124]													
PVA		2 [109,110]				1 [102]	2 [101,106]	1 [108]	1 [107]	2 [103,105]												1 [104]					
Gellan G		1 [131]			1 [130]	1 [129]	1 [127]				1 [128]																
SF							4 [31,132,133,134]	1 [135]										1 [136]									
Agarose			1 [139]		1 [138]						1 [137]																
PAMPS PDMA				2 [140,141]	1 [142]																						
Collagen								1 [144]			1 [143]																
CS		1 [146]						1 [145]																			
PAAm										1 [147]			1 [148]														
PAA										1 [149]																	
PNAGA THMMA																											1 [150]
PLGA-PBE													1 [151]														
Polyglucosamine													1 [152]														
ELR-based							1 [153]																				
HCF									1 [154]																		
Frequency	6	18	12	7	17	2	12	9	5	8	13	5	9	2	2	3	-	1	-	-	1	1	-	-	-	-	1
%	4.5	13.4	9.0	5.2	12.7	1.5	9.0	6.7	3.7	6.0	9.7	3.7	6.7	1.5	1.5	2.2	-	0.7	-	-	0.7	0.7	-	-	-	-	0.7
Frequency	36			26			26			26			13			4			1			1			1		
%	26.9			19.4			19.4			19.4			9.7			3.0			0.7			0.7			0.7		

This table summarizes the literature search results for the techniques utilized to process each hydrogel. H: homogeneous, L: layered, G: graded. Numbers for each process and hydrogel represent the frequency of publication relevant to the said hydrogel and process. The numbers in parentheses represent the references.

**Table 2:** Summary of in vivo osteochondral defect models.

Animal Model	Defect Location	Defect Size (mm)	In Vivo Duration (Weeks)	Outcome Characterization Methods
Rat	Femoral trochlea; femoral condyle	2–4	6–24	Histology (H&E, Safranin-O/Fast Green), IHC (COL I, II, X), micro-CT, biomechanical test
Rabbit	Medial femoral condyle; trochlear groove	2–6	4–24	Histology, IHC (COL II, aggrecan), macroscopic scoring, micro-CT
Pig/Minipig	Femoral condyle; trochlea	4–8.5	16–24	Histology, IHC, micro-CT, MRI, biomechanical test
Horse	Femoral condyle	10	26–48	Histology, IHC, micro-CT, MRI, biomechanical test
Dog	Femoral condyle	6	12	Histology, IHC, imaging, biomechanical test
Sheep	Femoral condyle	8	16	Histology, IHC, micro-CT, biomechanical test

## Data Availability

No new data were created or analyzed in this study. Data sharing is not applicable to this article.

## Supplementary Materials

The following supporting information can be downloaded at: https://www.mdpi.com/article/10.3390/gels12010024/s1, Table S1: Frequency of the use of hydrogel types and their application in in vitro and in vivo studies.
