# Supplementary material for: Hydrogels for Osteochondral Interface Regeneration: Biomaterial Types, Processes, and Animal Models
Source: Gels. 2025 Dec 27;12(1):24. doi: 10.3390/gels12010024 (PMC12841089; doi:10.3390/gels12010024)
Supplement: Supplementary file 1 [file gels-12-00024-s001.zip › gels-4059286-supplementary.pdf]

Table S1. Frequency of the use of hydrogel types and their application in in vitro and in vivo studies.

Hydrogels	Articles	In Vitro	In Vivo	In vivo animal models							Ex vivo
				**Rabbit**	**Rat**	**Pig**	**Horse**	**Dog**	**Human**	**Sheep**	**(Cadaver)**
**Gelatin**	36	6	30	20	8	2	-	-	-	-	-
**Alginate**	18	5	13	4	6	2	-	-	-	1	-
**Chitosan**	14	5	8	5	1	-	1	1	-	-	-
**Hyaluronic Acid**	10	-	10	5	3	-	1	-	1	-	-
**PVA**	10	1	8	7	1	-	-	-	1	-	1
**OPF**	9	1	8	6	-	2	-	-	-	-	-
**PEG**	8	3	5	2	3	-	-	-	-	-	-
**Gellan Gum**	5	1	4	2	2	-	-	-	-	-	-
**Silk Fibroin**	6	-	8	5	3	-	-	-	-	-	-
**Agarose**	3	2	1	1	-	-	-	-	-	-	-
**PAMPS/PDMAA**	3	-	3	3	-	-	-	-	-	-	-
**Collagen**	2	-	2	1	-	-	-	1	-	-	-
**Chondroitin Sulfate**	2	-	2	1	-	1	-	-	-	-	-
**PAAm**	2	1	1	1	-	-	-	-	-	-	-
**PAA**	1	-	1	1	-	-	-	-	-	-	-
**PNAGA/ THMMA**	1	-	1	-	1	-	-	-	-	-	-
**PLGA-PBE**	1	-	1	-	1	-	-	-	-	-	-
**PG**	1	-	1	-	-	-	-	-	1	-	-
**ELR based**	1	-	1	1	-	-	-	-	-	-	-
**HCF**	1	-	1	1	-	-	-	-	-	-	-
Total	**134**	**25**	**109**	**66**	**29**	**7**	**2**	**2**	**3**	**1**	**1**
Percent		**18.7**	**81.3**	**49.3**	**21.6**	**5.2**	**1.5**	**1.5**	**2.2**	**0.7**	**0.7**

OPF: Oligo (poly(ethylene glycol) fumarate); PEG: Poly(ethylene glycol); PVA: Polyvinyl alcohol; PAMPS: poly-(2-Acrylamido-2-methylpropanesulfonic acid); PDMAA: poly-(N,N′-dimethyl acrylamide); CS: Chondroitin sulfate; PAAm: Polyacryl amide, PAA: poly(amino acid); PNAGA: poly(N-acryloyl glycinamide); THMMA: [tris(hydroxymethyl)methyl] acrylamide; PGA: Poly(glutamic acid); PLGA: poly(L-glutomic) acid; phenylboronate ester (PBE); PG: Polyglucosamine; ELR: elastin-like recombinamers; HCF: Heparin-conjugated fibrin.
